# Sketches of Brazil, Including New Views on Tropical and European Fever, with Remarks on Premature Decay of the System, Incident to Europeans on Their Return from Hot Climates

**Published:** 1852-10

**Authors:** 


*Sketches of Brazil, including new views on Tropical and European Fever, with remarks on premature decay of the system, incident to Europeans on their return from hot climates.* By Robert Dundas, M. D., Physician to the Northern Hospital, Liverpool, &c. &c. 12mo. pp. 449. London: John Churchhill, 1852.

This volume is offered to the profession as the fruit of the observation and experience of a long medical career, passed in various tropical countries throughout the world. The author, Dr. Dundas, was attached to the British army in the Peninsula, West Indies, and coast of Africa, and was for twenty-three years medical superintendent of the British hospital at Bahia, in Brazil. His opportunities, therefore, on the important topic of which he treats, have been ample; and, while we must express our dissent from his opinions as to "the essential identity of fever in all countries," we most cordially bear testimony to the interesting and able manner in which he has discussed the subject, as well as to the soundness of his views on the therapeutics of malarious fevers generally.

Dr. Dundas has adopted the lecture form of publication—his book professing to be "a series of lectures delivered at the Northern Hospital, (at Liverpool,) in the beginning of the present year." We have frequently expressed our distaste for the rambling and discursive style of writing for which this lecture form is usually an apology; and we must say that it appears particularly inappropriate in a controversial book like the one before us.

The two first lectures of the course are devoted to the causes and treatment of what the author terms the "break up" of health, so prevalent in Europeans, upon return from a protracted residence in tropical climates. Much of this he attributes to the comparative suspension of the function of the kidneys, which results from the temperature of hot climates; and, probably, with reason. Hepatic derangement, however, we are disposed to consider as the "fons et origo mali" here: the point, though, wants, in this country, the practical interest with which it is invested in Great Britain, owing to her numerous tropical colonial dependencies.

The gist of the book is an attempt to establish the unity of idiopathic fevers. The received distinction between periodical and continued fevers is rejected by Dr. Dundas, who maintains that the "law of periodicity or the disposition to remission and exacerbations, at certain intervals, will be *found to apply more or less distinctly to all human diseases*—to those arising in the most opposite conditions of the animal economy, and determined by morbific agents apparently the most dissimilar and opposite in their nature as well as in their results." From this starting point the author proceeds to detail and examine a variety of facts which came under his notice, in reference to the different forms of fever; whence he reaches the conviction,

"That fever is the expression of a type of disease essentially one and uniform, but admitting of an almost endless variety of forms; the simplest being displayed in the single paroxysm of an ague. It is furthermore obvious," he says, "on the most cursory observation, that the description of fever as a disease has, in the generality of instances, been drawn for certain localities, and not from the whole group of febrile diseases, as witnessed in different parts of the world. The typhus fever of Great Britain is superseded by the bilious remittent and intermittent in Southern climates—by the plague in the Levant—and by the yellow fever within the tropics. Each of these maladies, under the special influence of climate, temperament, different modes of living, and numerous other agencies, affects certain peculiarities in its progress; but they are all distinctly impressed by the phenomena universally characteristic of fever as a genus of disease in every clime."

"Acting on the conviction of the essential identity of the remittent and intermittent fever of the tropics, with the typhus fever of Europe, and aware of the specific action of quinine in *every stage* of the former diseases, I have resorted," he says, "to its administration in the ordinary typhus of this country and Great Britain in all its stages, and commonly with the happiest results."

And, indeed, Dr. Dundas appeals to the efficacy of quinine in controlling the typhus fever of Great Britain,—*equal* (he thinks) to its specific influence over the fevers of tropical climates—as a strong argument in favor of his views.

"I would ask," he says, "whether, irrespective of all other evidence, the specific power exercised by proper doses of quinine, over all these several forms of fever, does not afford conclusive proof that, in their essential nature, these fevers are identical, and differ only in form and degree."

These opinions are not without partizans in our own country, and certainly may challenge a plausible array of facts in their support. For ourselves, however, we believe that the fevers known as typhus and typhoid, (into the distinction between which we shall not now enter,) and those of a periodical type—intermittent, remittent, congestive, and, we are disposed to add, yellow—are essentially distinct in origin and pathological characters, and should be also distinct in treatment. The few facts, which apparently militate against such a distinction, seem to us readily explicable by supposing the occasional simultaneous development of more than one form of fever. And against the doctrine of *identity*, there is so much to be urged on the score of difference of climate, season, &c., under which the different types of fever occur, (without dwelling upon their well-marked pathognomonic distinctions,) that we cannot for a moment hesitate in rejecting it. As regards Dr. Dundas' *practical* point of identity—the assumed equal value of quinine in the treatment of typhus and periodical fevers—we should be content to try the question by it alone, "irrespective of all other evidence." In the United states, at least, we are satisfied that there will be a nearly unanimous denial of the *specific* efficacy of quinine, in controlling or cutting short typhus fevers. As a corroborant and supporter of the system, its great value in such fevers is of course admitted. But we cannot at all concede to it the same specific febrifuge influence over typhus as over intermittent, remittent, &c.

We have already adverted to Dr. Dundas' sound therapeutic views as to the *early* administration of large doses of quinine, in the treatment of periodical fevers. His book contains also interesting particulars on the medical topography of Bahia, including a notice of the incurable elephantiasis which prevails there.

